# Breast Cancer Screening during COVID-19 Emergency: Patients and Department Management in a Local Experience

**DOI:** 10.3390/jpm11050380

**Published:** 2021-05-06

**Authors:** Francesca Maio, Daniele Ugo Tari, Vincenza Granata, Roberta Fusco, Roberta Grassi, Antonella Petrillo, Fabio Pinto

**Affiliations:** 1 Department of Radiology, Marcianise Hospital, Caserta Local Health Authority, Viale Sossietta Scialla, 81025 Marcianise, Italy; francescamaio9@gmail.com (F.M.); fpinto1966@libero.it (F.P.); 2 Department of Breast Radiology, Caserta Local Health Authority Dictrict 12, Viale Paul Harris 79, 81100 Caserta, Italy; medicina@danieletari.com; 3 Department of Radiology, Istituto Nazionale Tumori IRCCS Fondazione G.Pascale di Napoli, Via Mariano Semmola 53, 80131 Naples, Italy; v.granata@istitutotumori.na.it (V.G.); r.fusco@istitutotumori.na.it (R.F.); 4 Department of Radiology, Università degli Studi della Campania “Luigi Vanvitelli”, Piazza Miraglia, 80138 Naples, Italy; robertagrassi89@gmail.com

**Keywords:** COVID-19, breast cancer screening, guideline, breast cancer, screening, pandemic

## Abstract

Background: During the COVID-19 public health emergency, our breast cancer screening activities have been interrupted. In June 2020, they resumed, calling for mandatory safe procedures to properly manage patients and staff. Methods: A protocol supporting medical activities in breast cancer screening was created, based on six relevant articles published in the literature and in the following National and International guidelines for COVID-19 prevention. The patient population, consisting of both screening and breast ambulatory patients, was classified into one of four categories: 1. Non-COVID-19 patient; 2. Confirmed COVID-19 in an asymptomatic screening patient; 3. suspected COVID-19 in symptomatic or confirmed breast cancer; 4. Confirmed COVID-19 in symptomatic or confirmed breast cancer. The day before the radiological exam, patients are screened for COVID-19 infection through a telephone questionnaire. At a subsequent in person appointment, the body temperature is checked and depending on the clinical scenario at stake, the scenario-specific procedures for medical and paramedical staff are adopted. Results: In total, 203 mammograms, 76 breast ultrasound exams, 4 core needle biopsies, and 6 vacuum-assisted breast biopsies were performed in one month. Neither medical nor paramedical staff were infected on any of these occasions. Conclusion: Our department organization model can represent a case of implementation of National and International guidelines applied in a breast cancer screening program, assisting hospital personnel into COVID-19 infection prevention.

## 1. Introduction

On 30 January 2020, the World Health Organization (WHO) officially declared the COVID-19 (coronavirus disease ‘19) epidemic, caused by the virus SARS-CoV-2, a public health emergency and then, on 11 March 2020, officially declared the global situation as a pandemic [1,2]. WHO data report 83 million confirmed cases worldwide since the start of the outbreak and 1,8 million deaths (data as of 5 January 2021). As of 7 January 2021, 2,220,000 cases, including 77,291 deaths, had been confirmed in Italy and reported to the WHO [2,3]. One of the predominant transmission mechanisms of the virus is through droplet particles. Other transmission mechanisms include contact with infected surfaces touched by people who, without a sufficient disinfection of the hands, then touch their own mouth, nose or eyes [1]. People are often infectious 2–3 days before they exhibit symptoms [4], so the proportion of pre-symptomatic transmission ranges from 48% to 62% [4]. Spreading by asymptomatic carriers is estimated at 25% [5]. Moreover, the use of symptoms-based screening does not alone provide protection for all people [5].

At the same time, routine breast imaging, such as a mammogram or a breast ultrasound (US) examination, requires very close contact with patients with no chances for physical distancing. When performing these exams, the patient’s face may be as close as 20–30 cm to the face of the radiologist and/or the radiographer performing the study [6]. Similarly, during US, stereotactic and MRI-guided breast procedures, including biopsies, drainages and clip placements, the interventional radiologist may be distanced at only 30 cm from the patient’s face. In fact, it has been reported that the risk of infection with the novel coronavirus progressively increases with physical proximity and prolonged contact with people with COVID-19 [6,7].

Unfortunately, during the outbreak, breast screening activities were interrupted, whereas only emergency cancer-related medical activities were performed. Since June 2020, as the severity of the disease’s infection rate reduced in our country, screening activities have been resumed. This raised the need for a protocol to guide specialists on measures to prevent COVID-19 infection and to optimize resources with the aim of ensuring the best service level in breast cancer screening. Accordingly, the aim of the present study was to propose a protocol for managing our daily screening activities in order to ward mitigate infection spread.

## 2. Methods

Our department operational plan was based on the master opinion of three radiologists, members of the Italian Society of Radiology and Interventional Radiology (SIRM), which are routinely involved into Italian National Healthcare Service. They identified two different categories of patients referred to the Radiology Breast Unit Department and depicted four possible clinical scenarios.

The department operational plan was drafted following both the national and international guidelines for COVID-19 prevention and those for breast unit organization. Moreover, the plan is supported by a comprehensive literature comprising relevant articles searched using the mesh terms “COVID-19 OR SARS-CoV 2” AND “Screening” AND “Management” AND “Breast Imaging”.

**Figure 1:**
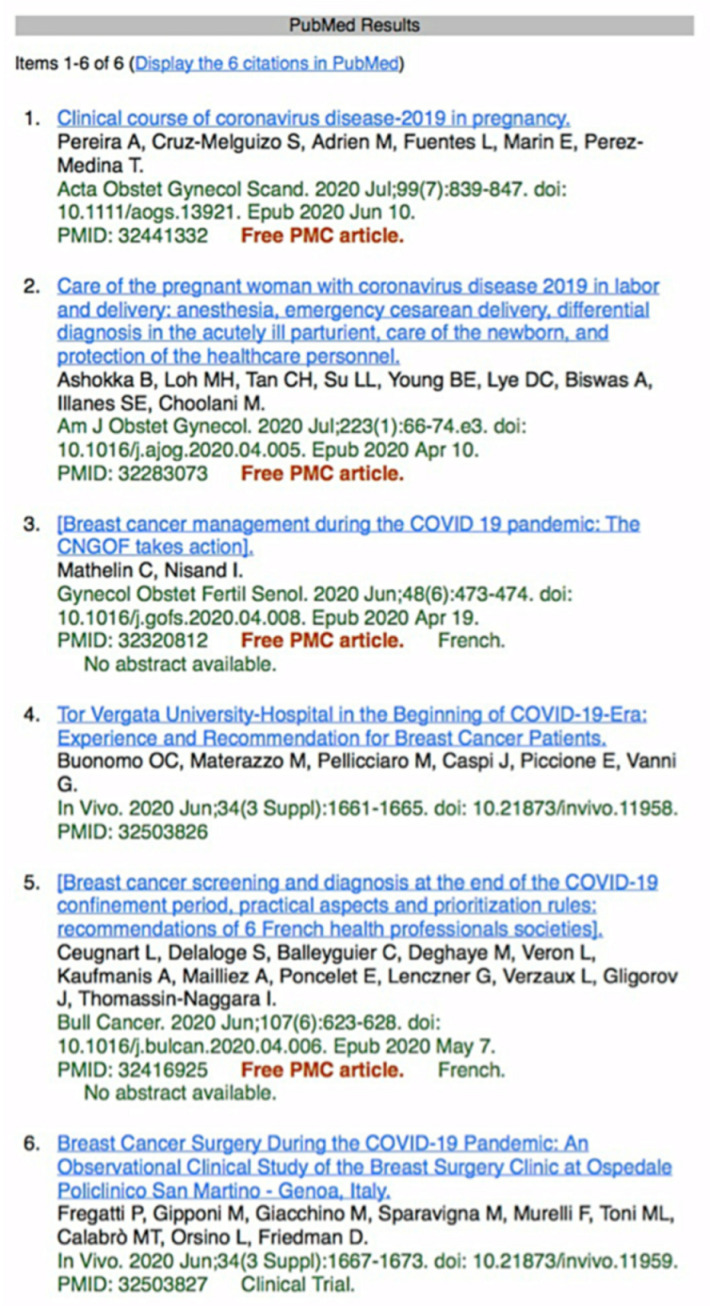
PubMed literature search using the mesh terms “COVID-19 OR SARS-CoV 2” AND “Screening” AND “Management” and “Breast Imaging” [8].

Overall, 6 articles, published between March and July 2020, were selected based on their relevance with respect to the primary endpoint (Figure 1). A summary of key findings was created for each of the relevant articles.

According to the essential levels of care [9,10], these are the two kinds of patient categories which attend the radiology breast imaging department:

Outline of Patients Categories

1. Breast screening patients:
  (a) Asymptomatic patients who undergo mammography exam following the specific screening program, according to national regulations.
  (b) Patients with suspected breast lesion revealed through the mammographic exam, thus needing to complete the work-up with ultrasound and needle biopsy.
2. Breast ambulatory patients:
  (a) Patients who have to complete the mammography work-up with ultrasound, following the surgeon recommendation.
  (b) Symptomatic breast cancer patients (new onset palpable nodule; skin or nipple retraction; orange peel skin; unilateral secretion from the nipple).

During the COVID-19 emergency for each of the above categories of patients, the following clinical scenarios can be delineated:

Outline of Clinical Scenarios

● Non-COVID-19 patient;
● Confirmed COVID-19 in asymptomatic screening patient;
● Suspected COVID-19 in symptomatic or confirmed breast cancer patient;
● Confirmed COVID-19 in symptomatic or confirmed breast cancer patient.

### 2.1. Practice Organization in the Radiology Breast Screening Department

Since the 3 June, when screening activities resumed, following the guidelines proposed by the SIRM Italian College of Breast Radiologists [11], the overall schedule of screening patients was split as follows: (1) patients who received a screening invitation before the COVID-19 pandemic onset within three months from the previous appointment were progressively scheduled; (2) symptomatic patients and those needing a needle biopsy for suspected cancer which were given an appointment with urgency (patients from group 1b and 2b, as shown above), were called back in order to complete the diagnostic pathway within 3 days; (3) ambulatory patients with previous appointments, were re-scheduled progressively for a dedicated day of the week. The time lapse between the exams was 30 min, with a total of 15 mammograms/ultrasounds per day. Moreover, a specific day per week was dedicated to ambulatory patients either for mammography or for breast ultrasound. Furthermore, a whole day was dedicated to breast interventional radiology, including core needle biopsy and vacuum-assisted breast biopsy. The “one-stop approach” was not applied in our department. The staff daily shift was organized as follows: 2 radiologists, 3 technicians and 1 nurse per shift, for a maximum of one shift (8 h) per day.

### 2.2. Infection Prevention in a Radiology Breast Screening Department

**Figure 2:**
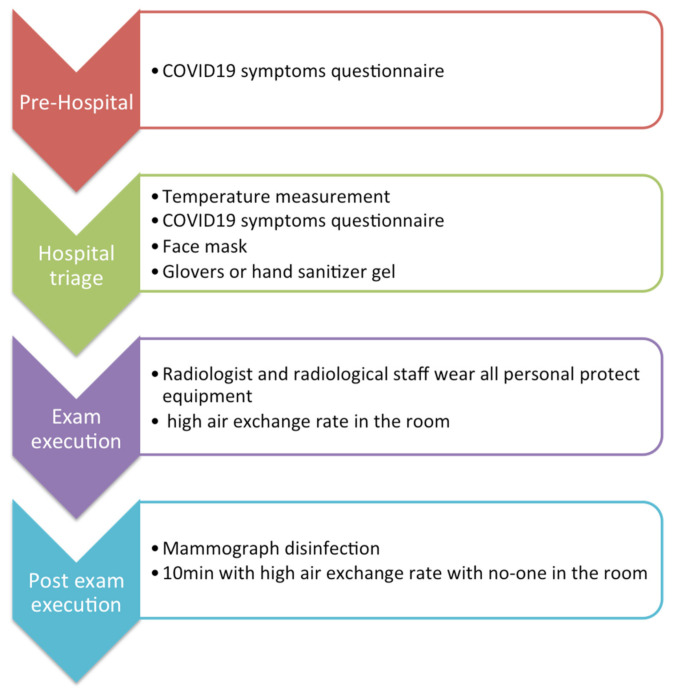
Patients and department management.

According to the recommendations of national legislation ISS COVID-19 n. 1/2020 [12] and the WHO recommendations set on February 2020 [2], which were properly adapted to our local requirements (refer to Figure 2), all patients had to undergo a telephone triage with a dedicated radiographer on the day before the radiologic exam. The pool of questions, fully reported in Table 1, was asked again and evaluated by the radiologist before the exam was performed. Body temperature measurement was performed for each patient before entering the hospital. Each appointment was scheduled every 30 min, in order to allow enough time for the exam execution, possible additional imaging (i.e., magnification views or spot views), and for the equipment’s disinfection and air ventilation (10 min). Patient capacity in the waiting room was set at a maximum of two people. An entrance and an exit door were designated, so to optimize the use of spaces and avoid interaction with subsequent patients. Moreover, to prevent the infection by SARS-CoV-2 of both medical and paramedical staff and other patients, more procedures could be adopted, depending on the different clinical scenario below:

● *Non-COVID-19 patient:* Patients without COVID-19 infection, as laboratory-confirmed by a reverse transcriptase-polymerase chain reaction (RT-PCR) test, were defined as non-COVID-19 patients [13]. However, since the laboratory tests had not yet been used as a screening tool to identify COVID-19 patients and many people may be asymptomatic or pauci-symptomatic, it would be appropriate for health professionals to consider all patients as if they were infected [14]. Therefore, all patients must wear a surgical mask and maintain the minimum distance of 1 m from others while waiting for a radiological procedure. No one, including any accompanying person, is allowed to stay in the waiting room. The healthcare staff should a wear surgical mask, avoid direct contact with patient’s oral and respiratory secretions, wear goggles or face shields and gloves and also wash hands before wearing and after removing gloves. A surgical cap and shoe covers are welcome. The ultrasound probe should be protected by a dedicated cover and disinfected after every single procedure [14,15,16]. After each radiological exam, the room and the radiological equipment must be cleaned and disinfected with chloro-derivate solutions and the room should be appropriately ventilated (>25 cycles/h) [14,17,18].
● *Confirmed COVID-19 in asymptomatic screening patient*: Considering the highly contagious nature of SARS-CoV-2, and taking count that this category of patients has no urgency to perform the mammographic exam, their appointments were rescheduled, as soon as was possible, after two negative nasopharyngeal swabs for SARS-CoV-2 RT-PCR test.
● *Suspected COVID-19 in symptomatic or confirmed breast cancer patient:* As in the first scenario, the patient must wear a surgical mask and follow the rules of social distancing in the waiting room. Radiological staff should wear an FFP2 mask (filtering face piece), goggles or face shield, gloves and cap. Ultrasound and mammographic machines must be covered by a plastic sheet and disinfected after the procedure with chloro-derivate solutions and the room should be appropriately ventilated (>25 cycles/h) [14,18].
● *Confirmed COVID-19 in symptomatic or confirmed breast cancer patient:* Considering the highly contagious nature of SARS-CoV-2, the patient wears a surgical mask and stays isolated from other people. Radiological staff must wear an FFP3 mask, eye protection, impermeable full-length long-sleeved gown, gloves and cap. Staff will pay maximum attention to the dressing and undressing procedures, as suggested by the Spallanzani Hospital [19]. Ultrasound and mammographic machines have to be covered by a plastic sheet and disinfected after the procedure with chloro-derivate solutions and the room should be appropriately ventilated (>25 cycles/h) [14,15,18].

Medical and paramedical staff were screened every month with a nasopharyngeal swab for SARS-CoV-2 RT-PCR test.

## 3. Results

Since 8 March 2020, 310 previously scheduled exams (267 breast screening and 43 breast ambulatory patients) were initially postponed because of the pandemic outbreak. Between 9 March and 29 May 2020, seven mammographic exams and two breast USs were performed for *ambulatory patients* identified as urgent/symptomatic for breast cancer. None of them resulted as confirmed breast cancer. One of them resulted as a suspected COVID-19 patient at the incoming triage.

Therefore, since the screening activities were resumed, those pending appointments were rescheduled, as soon as was possible, following the operational model above. In total, 205/267 previous appointments were rescheduled; 62/267 patients were no longer interested in having a screening appointment and were mostly scared by the SARS-CoV-2 infection risk. Some additional 50 new patients started regularly scheduling for breast cancer screening. In conclusion, 255 patients were screened in a month (3 June–3 July). Overall, 203 mammographic exams were performed, out of which 24/203 underwent a second-look US. In 52 cases, a breast US exam was performed as the first control. Moreover, four patients needed a core needle biopsy and six a vacuum-assisted breast biopsy: four patients were treated with subsequent surgery. No locally advanced breast cancer stage, such as: cancer >5 cm, or with skin/chest muscles infiltration, or multiple local lymph nodes invasion or a rapidly growing type [20], was revealed in the patients whose appointments had been postponed. All of the 255 patients who underwent breast cancer screening exams resulted as *non-COVID-19 patients* at the previous triage.

Until December 2020, 1479 *screening patients* received a mammographic exam. Of these patients, 163/1479 underwent a second-look US, 86 patients needed a core needle biopsy and 74 patients performed a vacuum-assisted breast biopsy. A total of 83 patients were treated with subsequent surgery. Overall, 15 *asymptomatic screening patients* resulted as *confirmed COVID-19 infections* at the telephone triage.

Moreover, in the same time interval, 174 *ambulatory patients* were screened by breast US. Among the 174 patients, 26 were symptomatic for breast cancer, out of which 9/26 received surgery for confirmed breast cancer. Among the 26, 5 symptomatic breast cancer patients were *suspected COVID-19* cases at previous triage. As of December 2020, no ambulatory patients were classified as *confirmed COVID-19* in symptomatic or confirmed breast cancer *patient.*

As for the matter of safety, a total of five radiologists, five technicians and one nurse were screened for SARS-CoV-2 between the beginning of March and the end of December, as described above. Each of them performed a total of 10 nasopharyngeal swabs. Nobody from the medical and paramedical staff resulted as positive to SARS-CoV-2 RT-PCR test.

## 4. Discussion

Our prospective study demonstrates efficacy in terms of continuity in the provision of an essential level of care in breast cancer screening. Furthermore, the absence of medical and paramedical staff SARS-CoV-2 infection is an additional fact that proofs the effectiveness of the infection prevention procedures adopted.

Due to the COVID-19 public health emergency outbreak, breast cancer units across the Italian territory have suffered significant restrictions and reductions in their clinical activities. Breast cancer is the first leading cause of cancer disease in the female population in Italy, with more than 50,000 breast cancer diagnosed every year and, out of which, 5000 are early breast cancer (infiltrating cancer <1 cm or ductal carcinoma in situ) [21]. The national screening program has improved the prognosis of patients with breast cancer by approximately 87% in 5 years, resulting in a lower number of tumors at the advanced stage (about 30%) [22]. The incidence rate reduction represents, also, a resource for our health system in terms of adjuvant therapy reduction, surgery duration, early return to work and improvement of the life quality standards. The estimated doubling time of breast cancer ranges between 45 and 260 days [23]. The latter growth rate variability did not allow us to estimate, precisely, the impact on not invited patients at breast cancer screening during the COVID-19 outbreak. A recent study compared breast unit activity in the first half of 2020 to the same time period on 2019 [24]. It reported an increased number of referrals either for diagnostic exams in suspected breast cancer patients (estimated around 28%) or for patients who received their first treatment for a breast cancer diagnosis (estimated around 16%) [24]. However, as reported in the literature [11], a short delay (e.g., 6–12 weeks) should not, in principle, affect the overall outcome. Furthermore, considering the periodical interruption/continuation of breast cancer screening activities, following the SARS-CoV-2 spread of infection in the population, these effects could be considerable on the female population. Vanni et al. [25] have estimated that 50% of the 11,000 cases will be identified with a delay of only 6 months, associated to a cancer stage progression. Moreover, they report that 8125 breast cancer diagnoses could be missed due to a screening interruption of 3 months [25]. This delayed diagnosis has several consequences, such as: an increase in the number of patients needing a diagnostic paths and treatments; a more invasive breast surgery or neoadjuvant or adjuvant therapy with a worse patient outcome; and an increase in healthcare costs. Therefore, some centers suggest a personalized screening program activity, which could be applied on urgent patients [22] or on patients with a high risk of breast cancer [26]. However, it is already known that its effectiveness in terms of incremental cost-effectiveness ratio (ICER) and quality adjusted life years (QALY) as well as its application during outbreaks could reduce their effect on women’s health [27]. Consequently, an optimized and effective department organization, which allows continuing screening and preserves the regular breast cancer-related medical activities, is achievable, especially in consideration of the unpredictable COVID-19 pandemic evolution. Moreover, continuing breast cancer screening during a pandemic emergency will avoid having to raise assistance requests at the end of the lockdown period [28].

To the best of our knowledge, national and international guidelines on breast cancer treatment in patients with SARS-CoV-2 infection have not yet been updated. Thus, nowadays, breast cancer patients with confirmed COVID-19 have to wait 10 days and two negative nasopharyngeal swabs before surgery [29], which may result in a worsened situation.

Some centers [28] suggest PCR testing before breast interventional procedures in patient with a BIRADS 5 lesion, such as to reduce the waiting time before surgery. However, this would increase the cost of each procedure, and additionally, the waiting time (from 20 min to 2 h) before each interventional procedure [30,31,32,33,34,35,36,37,38,39,40,41,42,43,44,45,46,47,48,49,50,51]. Consequently, it would increase the time lapse between the exams with a reduced number of procedures accomplished per day.

In our experience, our proposed model has proven a contraction of the waiting lists in a few weeks and also, has not been reported cases of advanced breast cancer stage.

Furthermore, a well standardized and SARS-CoV-2-free model is desirable to reduce the time lapse between the diagnoses and the treatment, avoiding the lengthening of waiting lists.

In conclusion, as little is known about the pandemic evolution, especially of its duration, the impact on screening breast cancer could be worse than reported. Therefore, our protocol, used to manage patients and radiological staff, could serve as a best practice in the application of national and international guidelines in the domain of the breast cancer screening program. If largely disseminated, it could assist specialists in preventing COVID-19 infection and in optimizing resources for breast cancer screening diagnosis.

## Figures and Tables

**Table 1:** Clinical assessment checklist the telephone questionnaire used to screen patients for COVID-19 infection before the radiological exam.

Questions	Yes	No
Do you have fever at the moment?		
Do you have cough?		
Did you have dyspnea or any respiratory disease, recently?		
Did you have anosmia or dysgeusia symptoms, recently?		
Did you have diarrhea, recently?		
Did you have unusual fatigue, recently?		
Did you have rash or ophthalmological disorders, recently?		
Have you done COVID-19 serology test?	If yes, ask for the test’s result	
Have you performed nasopharyngeal swab for COVID-19?	If yes, ask for the test’s result	

If any answer results positive, the patient will not be admitted to attend the radiological exam and rescheduled after a complete health evaluation made by his general practitioner doctor.

## Data Availability

The reported data come from SANIARP.it, the ASL Caserta reporting database and from the register of our daily activities.

## Abbreviations

WHO	World Health Organization
COVID-19	coronavirus disease ‘19
SARS-CoV-2	severe acute respiratory syndrome coronavirus 2
US	ultrasound
SIRM	Italian Society of Radiology and Interventional Radiology
MRI	magnetic resonance imaging
RT-PCR	reverse transcriptase-polymerase chain reaction
FFP	filtering face piece
BIRADS	breast imaging reporting and data system
